# Monitoring of pH Using an i-Motif-Forming Sequence Containing a Fluorescent Cytosine Analogue, tC

**DOI:** 10.3390/molecules24050952

**Published:** 2019-03-08

**Authors:** Patrycja Bielecka, Anna Dembska, Bernard Juskowiak

**Affiliations:** Faculty of Chemistry, Adam Mickiewicz University, Umultowska 89b, 61-614 Poznan, Poland; aniojka@amu.edu.pl (A.D.); juskowia@amu.edu.pl (B.J.)

**Keywords:** i-motif, fluorescent probe, fluorescent analogue tC, DNA, pH-probe

## Abstract

The i-motif is a four-stranded DNA structure formed from the cytosine (C)-rich ssDNA sequence, which is stabilized in slightly acidic pH. Additionally, labeling of a cytosine-rich sequence with a fluorescent molecule may constitute a way to construct a pH-sensitive biosensor. In this paper, we report tC-modified fluorescent probes that contain *RET*-related sequence C_4_GC_4_GC_4_GC_4_A. Results of the UV absorption melting experiments, circular dichroism (CD) spectra, and steady-state fluorescence measurements of tC-modified i-motifs are presented and discussed here. Efficient fluorescence quenching of tC fluorophore occurred upon lowering the pH from 8.0 to 5.5. Furthermore, we present and discuss fluorescence spectra of systems containing tC-modified i-motifs and complementary G-rich sequences in the ratios 1:1, 1:2, and 1:3 in response to pH changes. The fluorescence anisotropy was proposed for the study of conformational switching of the i-motif structure for tC-probes in the presence and absence of a complementary sequence. The possibility of using of the sensor for monitoring pH changes was demonstrated.

## 1. Introduction

In recent years, there is an increasing interest in developing new aptamers for the detection of any kind of target (small molecules, proteins, cells), both for diagnostic and basic research applications. DNA and RNA are attractive biomaterials for constructing self-secondary structures, such as aptamers and biosensors [1]. The i-motif (iM) structure represents an example of the structural elements used in biosensor construction [2,3,4,5]. I-tetraplexes are formed by C-rich sequences through intercalation of two parallel duplexes with the hemi-protonated base pairs. The building component of the i-motif structure is a base pair C-C^+^ with one cytosine protonated at the N3 position, which is stabilized in slightly acidic pH [6,7]. I-motif can be defined by the number of strands involved; an intermolecular i-motif forms from the interactions of two or four DNA strands, while an intramolecular i-motif assembles by folding of a single DNA strand [7,8]. Cytosine-rich tracts with the potential to form iMs are frequent in the telomeric DNA sequences [7] and in the promoter regions of oncogene sequences, such as *c-Myc* [9], *Rb* [10], *RET* [11], *VEGF* [12], and *Bcl-2* [13]. The main factors affecting the structure and stability of the i-motif structure beside pH are: temperature [8], ionic strength of the solution [8,14], presence of molecular crowding agents [15,16], and composition of the oligomeric strands. The ability of C-rich oligonucleotides to form i-motifs depends on the number of C-C^+^ base pairs [17,18], the length and nature of loops [19,20,21], the bases at the 5′ and 3′ ends of C-rich sequences [22,23], etc. It is well known that i-motif structures are predominant at acidic pH values with the conformational transition at pH ~6.5. However, the transition midpoint can be shifted as it strictly depends on the sequence and environmental conditions [24].

Fluorescent labels are frequently used for the modification of i-motif DNA sequences for sensing purposes [25]. Miscellaneous fluorescence parameters are exploited, for example quenching [26,27], anisotropy change [28], FRET (fluorescence resonance energy transfer) signal [29,30], or excimer emission signal [31,32]. In our approach reported already, the intramolecular i-tetraplex building oligonucleotide is modified by substitution of one cytosine with the fluorescent analogue of cytosine, tC [26]. The tricyclic family of cytosine analogues: tC (1,3-diaza-2-oxophenothiazine), tC^O^ (1,3-diaza-2-oxophenoxazine) and tC_nitro_ (7-nitro-1,3-diaza-2-oxophenothiazine), are derivatives of a phenothiazine or phenoxazine tricyclic framework [33]. The fluorescence quantum yield of tC is essentially unchanged when incorporated in single- and double-stranded DNA (0.17-0.24 and 0.16-0.21, respectively) irrespective of the neighboring bases [34,35]. Furthermore, tC has been used as a FRET-donor in a pair with rhodamine in a PNA–DNA hybrid [34], and in a pair with Alexa-555 in a study of conformational dynamics of DNA polymerase [36]. The 1,3-diaza-2-oxophenoxazine, tC^O^, is the brightest fluorescent base analogue when incorporated into duplex DNA (0.22) [37]. In the case of nitro-substitution, the tC_nitro_ is virtually nonfluorescent in polar solvents at room temperature [33]. The red-shifted absorption of tC_nitro_ has a considerable spectral overlap with the fluorescence spectra of both tC and tC^O^ and we can use the fluorescent analogues of cytosine as FRET pairs in nucleic acid studies (tC_nitro_ as a FRET acceptor and tC or tC^O^ as a donor) [38].

Additionally, the synthetic analogues of cytosine are very similar in both size and shape to the naturally occurring bases in DNA. Consequently, C-analogue incorporation into both single- and double-stranded DNA induces minimal perturbation to the overall structure [37]. Moreover, a characterization of the analogues showed that pH, salt, and temperature have very limited effects on their photophysical properties under biological conditions [33]. Reilly and coworkers showed that fluorescent 1,3-diaza-2-oxophenoxazine (tC^O^) analogue can be used to monitor individual substitution positions in an i-motif structure [27]. They used a 20-mer mutant strand labeled at five positions with individual tC^O^ substitutions (in C-C^+^ pair and in loop of i-motif). It has been demonstrated that folding of i-motif structure provided quenching of tC^O^ analogue fluorescence by hydrogen bonding and stacking interactions. Recently, we have demonstrated a new i-motif-switching aptamer containing a tricyclic cytosine analogue, 1,3-diaza-2-oxophenothiazine (tC) [26]. This sensor, based on *RET* proto-oncogene sequence, was substituted with tC at the 1st, 3rd, and 6th position from the 5′ end. The data showed that tC emission band underwent quenching upon lowering pH from 8.00 to 5.50. Moreover, the tC modifications of the i-motif structures show small differences in melting temperature between tC containing probes and unlabeled sequence.

Fluorescence anisotropy measures the rotational mobility of the fluorophores that are excited with polarized light (Equation (1)). Fluorescence anisotropy decay time constants are obtained by fitting the induced orientational anisotropy decay function, calculated from the experimental polarized transients. The anisotropy value is sensitive to the volume and structural changes of the fluorescent entity. Li and co-workers first used fluorescence anisotropy to study the structural switch of the i-motif structure [28]. This study demonstrated that in the presence of mismatches, the initial double helix was destabilized and an i-motif structure was observed accompanying the increase of the fluorescence anisotropy signal.

Very recently, Christ and co-workers provide evidence that i-motif structures are formed in regulatory regions of the human genome [39]. The authors developed an antibody (iMab) that recognizes i-motif structures with high selectivity and affinity, enabling the detection of i-motifs in the nuclei of human cells. The latter group demonstrated that the iMab antibody binds different well-defined i-motif structures while showing absence of binding to a wide range of control protein and nucleic acid antigens, including double-stranded DNA, hairpin DNA, microRNA, streptavidin, neutravidin, hen egg-white lysozyme, and neuropeptide Y (NPY). Moreover, using state-of-the-art in-cell NMR spectroscopy, Trantirek et al. showed that i-motif in vivo stabilities were generally distinct from those in vitro [40]. The data suggests that i-motif formation of DAP, PDGF-A, and JAZF1 takes place at physiological conditions. The results from these two studies [39,40] prove the existence of i-motif structures in vivo and indicate that i-motifs may have relevance in key biological processes.

In the current research, we utilize the tC analogue as a fluorescent nucleobase to detect the i-motif folding ability of a 20-mer *RET*-related sequence. We present here the spectral properties of these fluorescent oligonucleotide probes containing tC at the 8th, 14th, 17th, and 19th positions from the 5′ end. As reference we used a thymine-rich sequence with tC at 1st position. The UV-Vis, circular dichroism, and fluorescence emission spectra are reported, and the temperature stability of the i-motif structures adopted by probes (melting profiles) are discussed. To prove the sequence’s conformational change upon pH change, measurement of the i-motif probes in pH range 5.50–8.00 solutions were tested. Moreover, we discuss the pH effect on the fluorescence spectra of tC-modified i-motifs with complementary G-rich sequences in the ratios 1:1, 1:2, and 1:3. The fluorescence anisotropy was proposed for the study of conformational switching of the i-motif structure in the presence and absence of a complementary sequence.

## 2. Results and Discussion

### 2.1. Design of tC Probes

**Figure 1:**
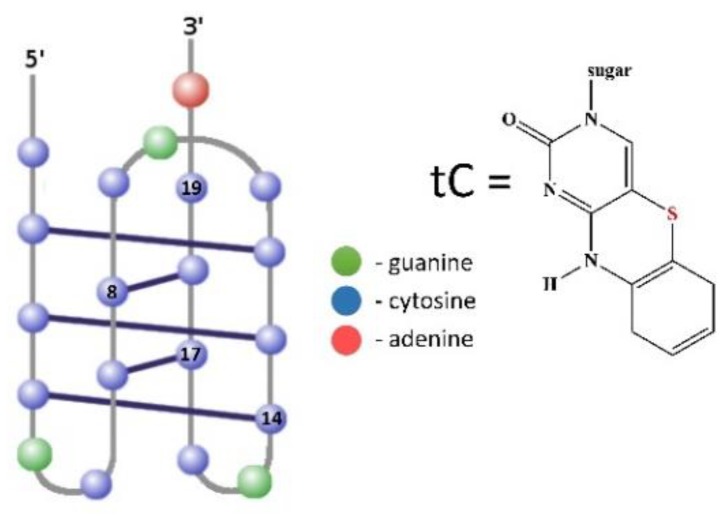
Structure of intramolecular i-motif formed by 8tC, 14tC, 17tC, and 19tC probes. The positions of the single fluorescent cytosine analog substitutions are numbered and blue circles—cytosines, green circles—guanines, and red circles—adenines.

The 20-mer fragment of the *RET* proto-oncogene was modified with fluorescent cytosine analogue tC (1,3-diaza-2-oxophenothiazine) by substitution cytosine at various positions. We designed three probes containing tC in the core of the i-motif (8th, 14th, or 17th position) and one probe with tC at the terminal position of the i-motif structure (19th position) (Table 1 and Figure 1). The oligonucleotide probes are named 8tC, 14tC, 17tC, and 19tC, respectively. As illustrated in Figure 1, tC in the 8th position is involved in base-pairing with 18th of cytosine, the 14th position is involved in base-pairing with 4th of cytosine, and the 17th position is involved in base-pairing with 7th of cytosine. However, the substitution of tC at the 19th position locates the fluorescent tag outside the i-motif structure. The reference sequence, TtC, contains the fluorescent cytosine analogue in 1st position and is composed mostly of thymine nucleobases. The T-rich strand is unable to form i-motif structure. The presented studies are a continuation of our research considering C_4_GC_4_GC_4_GC_4_A (RET20) substituted with the cytosine fluorescent analogue tC [26].

### 2.2. Thermal Stability of i-Motifs

**Figure 2:**
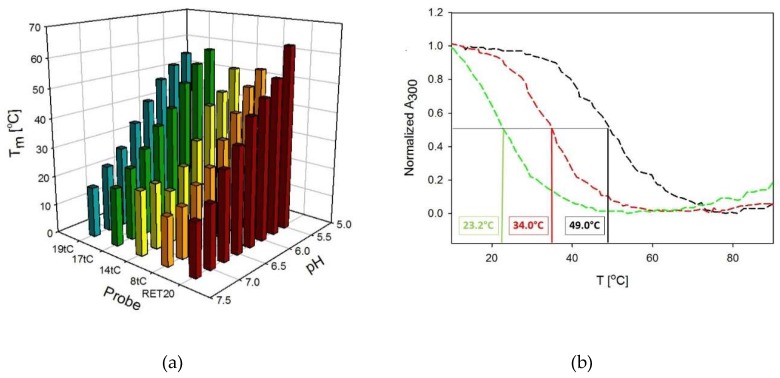
(**a**) Thermal melting temperatures for tC-modified probes: 8tC (orange), 14tC (yellow), 17tC (green), 19tC (blue), and unmodified 5′-(C_4_G)_3_C_4_A-3′ sequence (black) plotted against pH. All values and standard errors are presented numerically in Table 2; (**b**) Denaturation normalized profiles of the 8tC probe recorded at 300 nm at three different pH values: pH 5.75 (green dash line); pH 6.25 (red dash line); pH 6.75 (black dash line). The presented curves are obtained during heating.

Ultraviolet (UV) thermal melting was used to characterize the pH and thermal stability of the RET20 i-motif (unmodified sequence) and to assess the effect of tC substitution position (modified sequences). This method provided information about thermodynamic properties and different kinetics of folding and unfolding of i-motif structures. The thermal stability of each sequence was assessed in buffers with pH from 5.50 to 8.00. All T_m_ values were calculated from the denaturation profiles monitored at 300 nm and 260 nm. The obtained curves were Z-shaped at 300 nm and sigmoidal at 260 nm. Very similar results were obtained from the denaturation curves recorded at both wavelengths. As shown in Figure 2a, the thermal stability of the unmodified and modified RET20 structures were pH dependent. In the pH range 5.50–7.25, the Tm was a linear function of pH, as observed previously for a d((C_4_G)_3_C_4_A) sequences modified with fluorescent cytosine analogue tC at the 1st, 3rd, and 6th position from the 5′ end [26]. Decreasing the pH significantly increased the stabilization of the structure of RET20 i-motif, i.e., the T_m_ was 18 °C at pH 7.25, and increased to 63.4°C when the pH decreased to 5.50. At pH 7.50 and higher, RET20 as well as related probes can form lower-stability folded structures with T_m_ below 15 °C. The examples of thermal profiles are presented in Figure 2b for the 8tC probe at pH 5.75, 6.25, and 6.75, which were generated by the normalized absorbance versus temperature profiles. We evaluated the T_m_ values by curve fitting, and the T_m_ values for the presented melting curves were 23.2 °C, 34.0 °C, and 49.0 °C, respectively.

The unmodified RET20 oligonucleotide behaves very similar to the 17tC and 19tC probes. In the pH range 5.50–7.25, the differences in melting temperature (ΔT_m_) between RET20 and 17tC or 19tC are (5.0; −0.6; 0; 3.1; 0; 0.6; −3.2; −1.8) and (7.5; 1.1; 0.2; 2.4; 1.0; 2.8; −1.3; 0.8), respectively (Table 2). The trends in T_m_ found for these oligos suggest that the substitution of tC at the 17th and 19th position (at the 3′ end) leads to more stable i-motifs structures. Modification at the C8 and C14 positions resulted in lower stabilization of the structure compared to the other single tC-modified sequences. For example, the T_m_ of the 8tC probe is 9.3 °C and 9.9 °C degrees lower than in case of RET20 sequence in pH 5.50 and 6.25, respectively. Moreover, the same trend was observed in case of the 14tC, i.e., the T_m_ values were 10.0 °C and 12.3 °C degrees lower in pH 5.50 and 6.25 compared to those for the unmodified sequence. Bielecka and Juskowiak previously showed similar changes for i-motif probes substituted with tC at the 1st, 3rd, and 6th positions from the 5′ end [26]. Reilly et al. reported similar observation in thermal stability for the *c-myc* sequences substituted with the 1,3-diaza-2-oxophenoxazine (tC^O^) analogue [27].

### 2.3. CD Spectra

**Figure 3:**
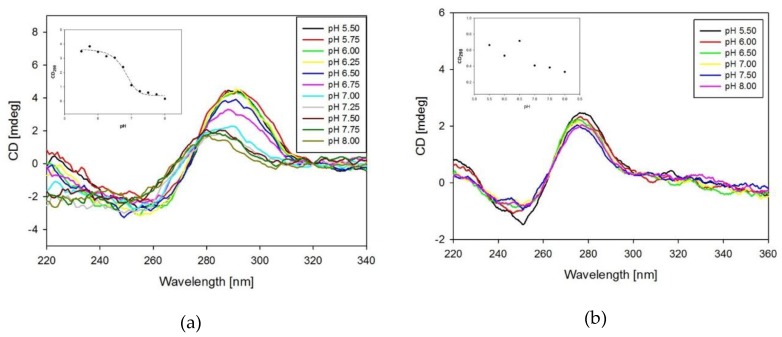
CD spectra of 17tC probe (**a**) and TtC probe (**b**) in the pH range of 5.50–8.00. Inserts show the dependences of CD signal at 288 nm against the pH values.

CD experiments were used to establish the presence of i-motif DNA structure in solution. In the pH range 5.50–7.00, the CD spectra of tC-containing probes exhibit a very sharp positive band at 288 nm and a weak negative band at 262 nm that is consistent with the spectral characteristics of the i-motif structure [9,11]. As an illustration, spectra for the 17tC probe are shown in Figure 3a. Furthermore, at pH values around 7.0, the positive maximum shifted toward 280 nm indicating partial unfolding of the i-motif structure. The positive peak at 280 nm sharply decreased with the increase in pH of the solution, possibly due to the deprotonation of C at alkaline or neutral pH values. Similar spectral signatures were observed for the unmodified structure (RET20) and other probes (Figures S1–S4 in Supplementary Materials). These results demonstrated that, after modification with fluorescent cytosine analogue tC, the resulting i-motifs still responded to pH changes and were able to perform conformational changes. CD spectra measured for the reference TtC probe (Figure 3b) indicate the lack of the characteristic signals observed for 17tC (Figure 3a), confirming the absence of i-motif formation for this reference sequence.

For each probe, CD signals observed at 288 nm were plotted against pH and fitted into a sigmoidal function. The pH values of i-motif transition (pH_T_) for all sequences are given in Table 2 and the representative graph is shown in Figure 3a (insert). For example, RET20 (unmodified) has a pH_T_ of 7.1±0.2, while probes 8tC and 19tC have slightly lower pH_T_ of 6.8 and 6.9, respectively. In the case of the 14tC and 17tC probes, the pH_T_ values are even lower (6.7).

### 2.4. Fluorescence Properties of the tC-Modified Probes

**Figure 4:**
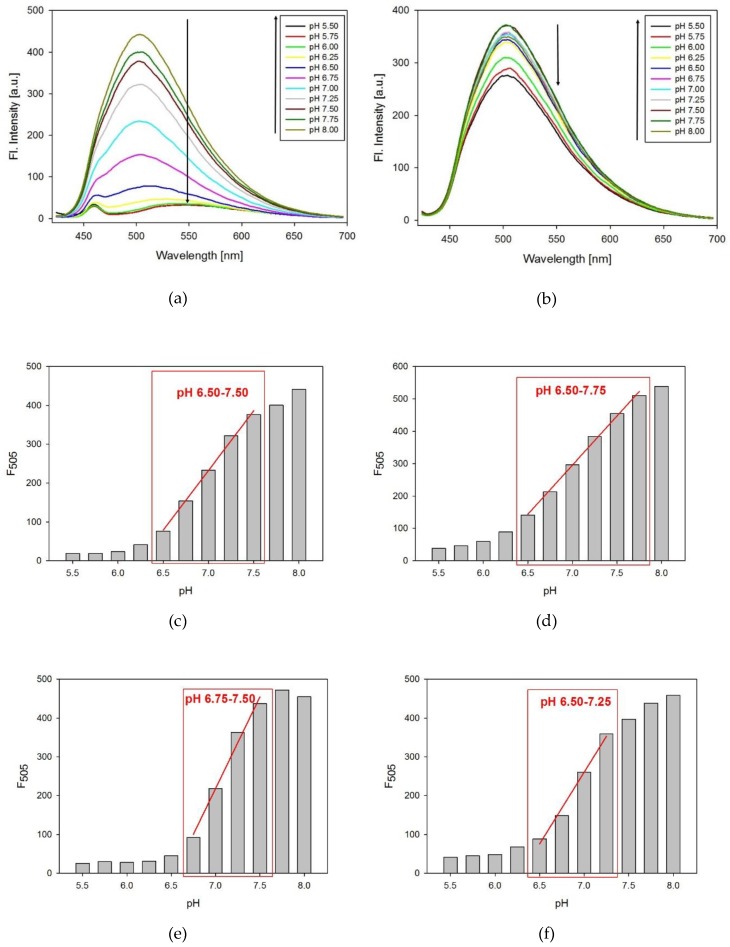
The emission spectra of the 8tC probe (**a**) and the TtC probe (**b**) in the pH range from 5.50 to 8.00. Panels (**c**–**f**) show pH working ranges for tC probes: 8tC (**c**), 14tC (**d**), 17tC (**e**), and 19tC (**f**). In all cases, the excitation wavelength was set at 395 nm.

The fluorescence spectra of the modified probes were measured as a function of pH. The tC fluorophore was excited at the maximum of absorption (395 nm). As shown on the example of 8tC (Figure 4a), the fluorescence intensity decreased with pH lowering from 8.00 to 5.50 and the emission band at 505 nm exhibited red shifting to ca. 540 nm. In all cases, lowering the pH caused substantial quenching and red shift in the emission band for acidic solution of i-motif probes (Figures S5–S7 in Supplementary Materials). In contrast, the reference TtC oligonucleotide at the same pH range exhibited much smaller reduction of fluorescence without any red shift (Figure 4b). To determine the changes in fluorescence intensity, we calculated ΔF (ΔF = (Int.F measured at pH 8.00 – Int.F measured at pH 5.50)/Int.F measured at pH 8.00) for each oligonucleotide probe. Three of the tC substituted probes (8tC, 14tC, and 17tC) exhibited very similar ΔF values: 0.954, 0.927, and 0.942, respectively. The probe with terminal substitution (at the 19th position) had a slightly lower ΔF value of 0.908. This result is consistent with the location of tC outside of the i-motif structure in the 19th position. Furthermore, the reference sequence, TtC, exhibited only a small change in fluorescence intensity with a ΔF value of 0.213 [26].

The pH working ranges for all probes were determined from the dependences of the fluorescence measured at 505 nm versus pH (Figure 4c–f). For the 14tC probe, the pH working range covers the widest range with a linear fluorescence variation between pH range from 6.50 to 7.75 (Figure 4d). Moreover, we observed the pH working range from 6.50 to 7.50 for the 8tC probe (Figure 4c). As shown in Figure 4e,f, two of the tC substituted probes (17tC and 19tC) exhibited the narrowest pH working ranges from pH 6.75 to 7.50 and 6.50 to 7.25, respectively. These results indicate that the position of substitution has an influence on the obtained pH working range and thus the probe’s sensitivity. At the same ΔF, a narrow pH working range gives the highest sensitivity.

### 2.5. Experiments with Complementary Strand (DNA Duplex and i-Motif Equilibrium)

**Figure 5:**
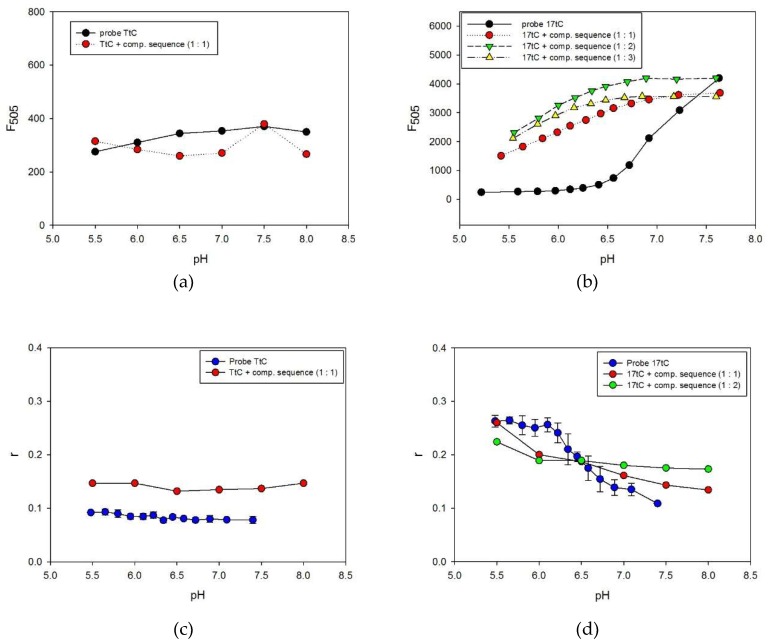
The dependence of fluorescence intensity at 505 nm vs. pH for: TtC (**a**) and 17tC (**b**) probes alone or with their complementary strand. Panels (**c**) and (**d**) show the dependences of fluorescence anisotropy vs. pH for TtC and 17tC, respectively, alone or with complementary strand.

All presented tC-modified probes exploited a ssDNA/i-motif transformation, which generated an analytical signal based on fluorescence quenching. The pH working range can be shifted through the tC substitution position (see above) but the magnitude of shift is limited. An alternative approach that may affect the working range of the probe is to exploit duplex/i-motif transformation, for example, as a Molecular Beacon platform. To characterize the fundamental processes in such systems, we tried to study i-motif probes in the presence of their complementary strand. The presence of the complementary strand to a C-rich sequence should affect i-motif folding, thus the fluorescence response of the probe will change. Fluorescence spectra were collected for each tC-substituted C-rich strand duplexed with the complementary G-rich strand in the pH range of 5.50–8.00. Additionally, we used different molar ratios of C-rich probes to complementary G-rich strand—1:1, 1:2, or 1:3. In order to clearly identify differences between particular systems, we plotted fluorescence intensity at 505 nm vs. pH and the results are shown in Figure 5a,b. The fluorescence intensities at 505 nm measured for TtC probe alone or with complementary A-rich strand (at 1:1 ratio) are very similar at all pH values (Figure 5a). Representative results for the 17tC probe are shown in Figure 5b. As expected, the i-motif probe in the absence of complementary strand exhibited a dramatic increase in quenching of fluorescence at pH values below 7.0. For dsDNA-containing systems, the formation of the i-motif structure was gradually reduced in the same pH range, as suggested by less efficient fluorescence quenching. The profiles shown in Figure 5b indicate that tC embedded in the duplex exhibits fluorescence that is not quenched in lower pH. The system responds to the pH variation as a result of competition between formation of duplex and i-motif at lower pH (5.50–7.00).

To prove the assumption that observed fluorescence changes originated from duplex/i-motif competition, we carried out anisotropy measurements for these systems. The fluorescence anisotropy provides information on molecular orientation and mobility, and thus is sensitive to the volume and structural changes of the fluorescent species. Figure 5c shows the anisotropy value change for the TtC reference probe alone or with a complementary A-rich strand under the same conditions. As expected, the fluorescence anisotropy values for the TtC probe are low (~0.1) in the studied pH range of 5.50–8.00. However, the TtC oligo indicates a significant anisotropy increase upon addition of its complementary sequence in the ratio 1:1. The presence of the complementary A-rich strand leads to duplex formation with larger mass, which hindered the rotational diffusion rate of the macromolecule. As illustrated in Figure 5d, higher fluorescence anisotropy values (r~0.25) were observed for the 17tC probe alone in the slightly acidic pH, due to the folded i-motif structure and restricted rotation of tC. i-Motif formation affects the shape of the oligonucleotide, and thus changes the rotational diffusion of the macromolecule. At alkaline or neutral pH values, the probe exists mainly in the flexible ssDNA form (tC free to rotate) with a decreased anisotropy signal comparable to that of TtC (r~0.1). Again, we have observed higher anisotropy signals for duplexes compared with ssDNA. Next, the i-motif-forming 17tC probe was hybridized with its complementary strand, forming a rigid duplex (dsDNA). Upon addition of acid, the solution pH decreased, leading to the folding of the i-motif structure and the release of the G-rich complementary strand. As result, the fluorescence anisotropy signal increased and can be successfully exploited as an indication of i-motif formation. However, in the presence of the complementary oligonucleotide at a ratio of 1:2, we observed a less sensitive pH response than at the ratio of 1:1 and a higher concentration of [H^+^] was required to force the formation of the i-motif structure. Therefore, a lower fluorescence anisotropy value was observed for the 17tC probe in the presence of its complementary strand (ratio 1:2) than for 17tC alone in solution pH 5.5 (Figure 5d). All these results indicated that it is possible to design a pH probe with fluorescent tC analogue, which is based on duplex/i-motif competition and enables programming of the pH working range.

## 3. Materials and Methods

### 3.1. Materials

The DNA oligonucleotides modified with fluorescent cytosine analogue tC (1,3-diaza-2-oxophenothiazine) (Figure 1 and Table 1) were purchased from Future Synthesis (Poznan, Poland). All oligonucleotides were synthesized and purified by Future Synthesis using a high-performance liquid chromatography (Prominence HPLC) (Shimadzu, Kyoto, Japan). Single-strand concentrations were determined by measuring the absorbance (260 nm) at high temperature (85 °C), assuming the molar extinction coefficients of 7400 M^−1^cm^−1^ for cytosine, 15,400 M^−1^cm^−1^ for adenine, 11,500 M^−1^cm^−1^ for guanine, and 4000 M^−1^cm^−1^ for analogue tC. Before spectral measurements, the DNA probes were annealed by being heated to 90 °C and then slowly cooled to room temperature (RT). Each probe was dissolved in 10 mM cacodylate buffer at concentration of 1.0 μM. For duplex formation, the mixture of probe and complementary strand (at ratios 1:1, 1:2, 1:3) was incubated at 90 °C temperature for 10 min and slowly cooling down to RT. All experiments were performed in 10 mM sodium cacodylate buffer with pH ranging from 5.50 to 8.00. Spectroscopic measurements were done in triplicate.

### 3.2. DNA Melting Temperature (T_m_)

Melting profiles were recorded on a spectrophotometer model Carry 100 (Agilent Technologies, Mulgrave, VIC, Australia) equipped with a Peltier temperature control accessory. UV melting curves were measured by monitoring the absorbance at 260 nm and 300 nm while the temperature was increased at a rate of 1 °C/min. The melting temperatures (T_m_) were determined as the maximum of the first derivative of the melting curves. Each T_m_ value was an average of three independent measurements.

### 3.3. Circular Dichroism (CD)

Circular dichroism spectra were recorded at 25 °C on a Jasco J-715 spectropolarimeter (Jasco, Tokyo, Japan) equipped with a Peltier temperature control accessory, typically using a scan rate of 200 nm/min over the wavelength range of 220–360 nm. The buffer spectrum was subtracted from the sample spectra.

### 3.4. Fluorescence Emission Spectra

Steady-state fluorescence measurements were recorded by a Cary Eclipse spectrofluorometer (Agilent Technologies, Mulgrave, VIC, Australia) at room temperature in the spectral range of 420–700 nm. All fluorescence measurements were performed under the same spectral conditions: the slit width for excitation was 10 nm and that for emission was 5 nm, and the excitation wavelength was set at 395 nm.

### 3.5. Fluorescence Anisotropy Measurements

Each sample solution was titrated with a solution of hydrochloric acid (0.5 M), followed by stirring and thermal equilibration. Steady-state fluorescence anisotropy was measured using a Cary Eclipse spectrofluorometer (Agilent Technologies, Mulgrave, VIC, Australia) with 395 nm excitation at room temperature.

The anisotropy, *r*, of the tested solution was calculated using equation (1):

(1) $$r=\frac{\left(I_{VV}I_{HH}-I_{VH}I_{HV}\right)}{\left(I_{VV}I_{HH}+2I_{VH}I_{HV}\right)}$$

where *I* represents the intensity of the fluorescence signal and the subscript defines the orientation (*H* for horizontal and *V* for vertical) of the excitation and emission polarizers, respectively.

## 4. Conclusions

We have designed four new pH-sensitive probes based on the d((C_4_G)_3_C_4_A) sequence expected to form i-motif structures. All cytosine-rich sequences contained the single cytosine fluorescent analogue 1,3-diaza-2-oxophenothiazine (tC) substituted at 8th, 14th, 17th, or 19th position from the 5′ end. We showed that tC substitution in the 8th or 14th position of iM results in lower T_m_ compared with the unmodified sequence, RET20, especially in the pH range of 5.50–6.75. On the other hand, the melting temperatures for 17tC and 19tC demonstrated that such modification did not affect the thermal stability compared with the d((C_4_G)_3_C_4_A) sequence. The pH_T_ values determined using CD spectroscopy suggest that the substitution of cytosine by fluorescent analogue at the chosen positions has a significant impact on i-motif folding. The emission band underwent gradual quenching upon lowering the pH from 8.0 to 5.5 in all cases. However, the pH working ranges determined for tC probes depend on the location of 1,3-diaza-2-oxophenothiazine (tC) in the C-rich sequence. The fluorescence anisotropy measurements demonstrated that destabilization of the initial i-motif structure at higher pH resulted in the lower initial fluorescence anisotropy value. On the other hand, the anisotropy studies upon duplex/i-motif competition revealed that iM exhibited a higher anisotropy signal than dsDNA which can be attributed to the smaller rotational freedom of a compact i-motif structure. These studies may be very useful in terms of designing molecular logic gates or more complicated biosensors, i.e., molecular beacons integrated with i-motifs.

## Figures and Tables

**Table 1:** Oligonucleotides used in this study and their respective sequences.

Name	Sequence 5′–3′ (Complementary Sequence 3′–5′)	Position of tC Analogue
RET20	CCC CGC CCC GCC CCG CCC CA	-
8tC	CCC CGC C**tC**C GCC CCG CCC CA	8
14tC	CCC CGC CCC GCC C**tC**G CCC CA	14
17tC	CCC CG**C** CCC GCC CCG C**tC**C CA	17
19tC	CCC CG**C** CCC GCC CCG CCC **tC**A	19
TtC	tCCC TTT TTT TTT TTT TTT TT	1
Complementary sequence to 8tC; 14tC; 17tC; 19tC	TGGG GCG GGG CGG GGC GGG G	-
Complementary sequence to TtC	AA AAA AAA AAA AAA AAA GGG	-

**Table 2:** The melting temperatures (T_m_) evaluated from denaturing profiles obtained at different pH values. pH_T_ determined from circular dichroism (CD) experiments.

Name	pH 5.50	pH 5.75	pH 6.00	pH 6.25	pH 6.50	pH 6.75	pH 7.00	pH 7.25	pH 7.50	pH_T_
RET20	63.4 ± 1.5	53.6 ± 1.3	48.5 ± 1.5	44.0 ± 1.4	36.1 ± 1.2	30.3 ± 1.1	21.5 ± 1.2	18.0 ± 0.8	<15	7.1 ± 0.2
8tC	54.1 ± 1.7	49.3 ± 1.2	41.7 ± 1.1	34.1 ± 1.3	26.5 ± 1.8	22.5 ± 1.0	17.5 ± 1.8	16.5 ± 1.5	<15	6.8 ± 0.2
14tC	53.0 ± 0.5	45.9 ± 0.7	42.4 ± 0.8	31.7 ± 0.6	24.4 ± 1.1	17.8 ± 2.6	22.3 ± 1.4	21.9 ± 1.8	<15	6.7 ± 0.2
17tC	58.4 ± 1.4	54.2 ± 0.7	48.5 ± 0.7	40.9 ± 0.9	36.1 ± 2.0	29.7 ± 1.2	24.7 ± 1.6	19.8 ± 0.9	<15	6.7 ± 0.2
19tC	55.9 ± 0.6	52.5 ± 0.6	48.3 ± 0.5	41.6 ± 0.6	35.1 ± 0.7	27.5 ± 1.2	22.8 ± 2.6	17.2 ± 2.5	<15	6.9 ± 0.2

## Supplementary Materials

The following are available online at https://www.mdpi.com/1420-3049/24/5/952/s1, Figure S1: CD spectra of RET20 sequences in the pH range of 5.50–8.00, Figure S2: CD spectra of 8tC probe in the pH range of 5.50–8.00, Figure S3: CD spectra of 14tC probe in the pH range of 5.50–8.00, Figure S4: CD spectra of 19tC probe in the pH range of 5.50–8.00, Figure S5: The emission spectra of 14tC probe at the pH range from 5.50 to 8.00, Figure S6: The emission spectra of 17tC probe at the pH range from 5.50 to 8.00, Figure S7: The emission spectra of 19tC probe at the pH range from 5.50 to 8.00.
