# Supplementary material for: Monitoring of pH Using an i-Motif-Forming Sequence Containing a Fluorescent Cytosine Analogue, tC
Source: Molecules. 2019 Mar 8;24(5):952. doi: 10.3390/molecules24050952 (PMC6429216; doi:10.3390/molecules24050952)
Supplement: Supplementary file 1 [file molecules-24-00952-s001.pdf]

Monitoring of pH using i-motif forming sequence containing fluorescent cytosine analogue, tC

Patrycja Bielecka[1,*], Anna Dembska[1] and Bernard Juskowiak[1]

1. Faculty of Chemistry, Adam Mickiewicz University, Umultowska 89b, 61-614 Poznan, Poland.;
* Correspondence: bieleckapat@gmail.com; Tel.: 48-61829-1771

Supplementary Materials

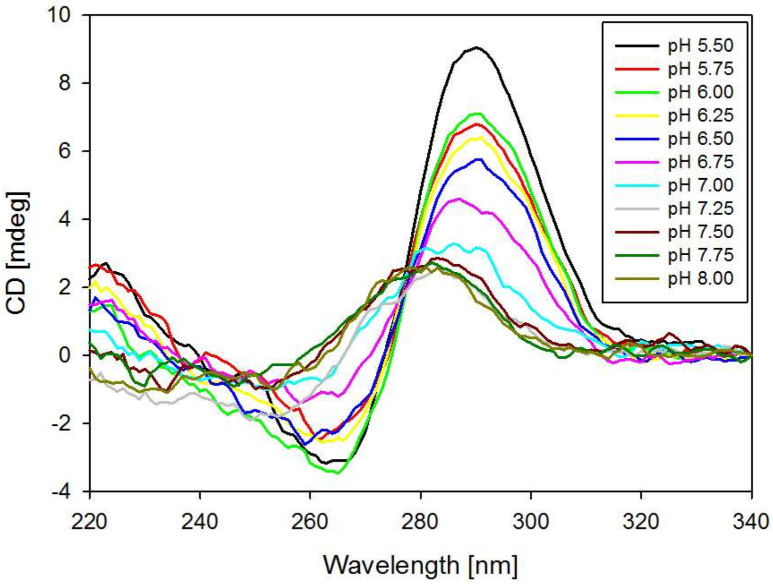

Figure S1. CD spectra of RET20 sequences in the pH range of 5.50–8.00.

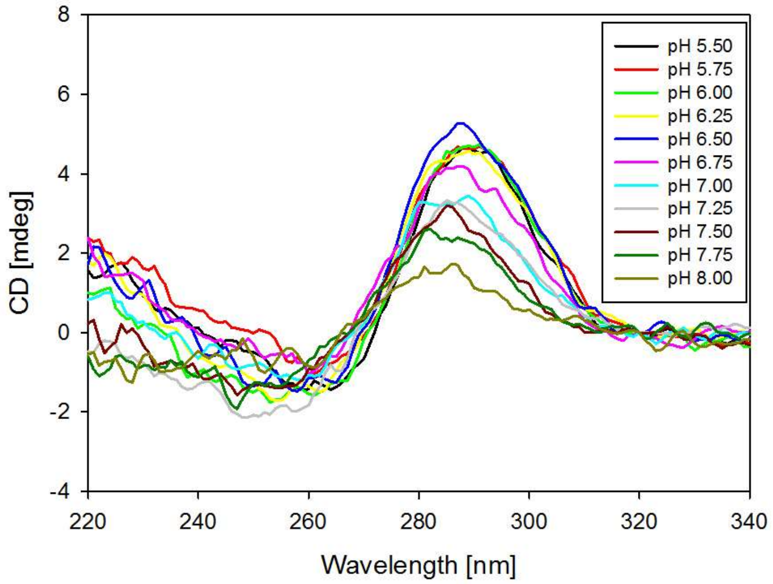

Figure S2. CD spectra of 8tC probe in the pH range of 5.50–8.00.

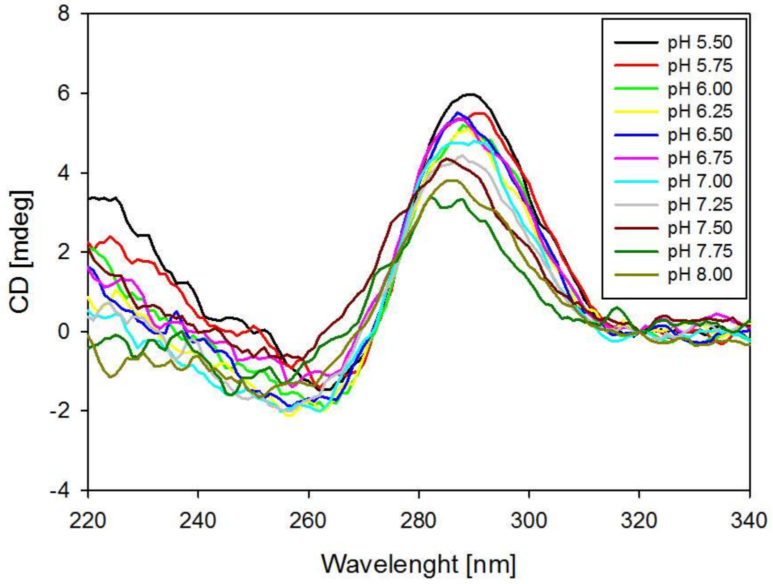

Figure S3. CD spectra of 14tC probe in the pH range of 5.50–8.00.

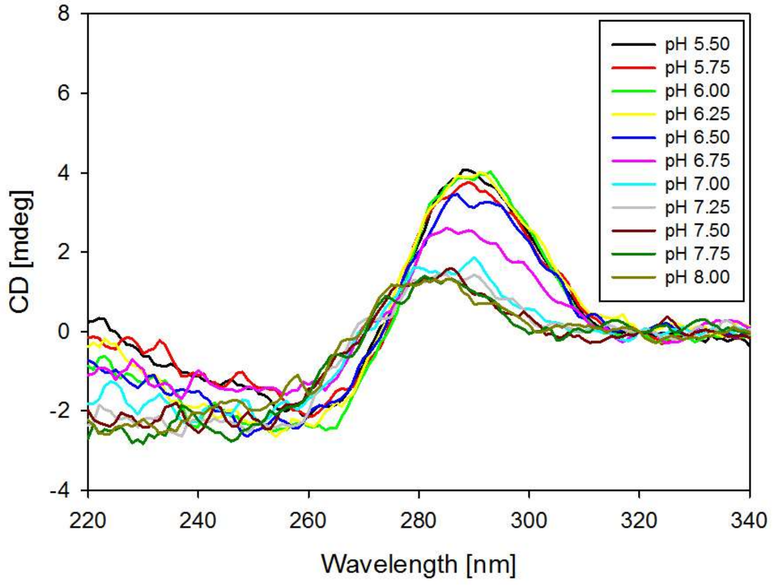

Figure S4. CD spectra of 19tC probe in the pH range of 5.50–8.00.

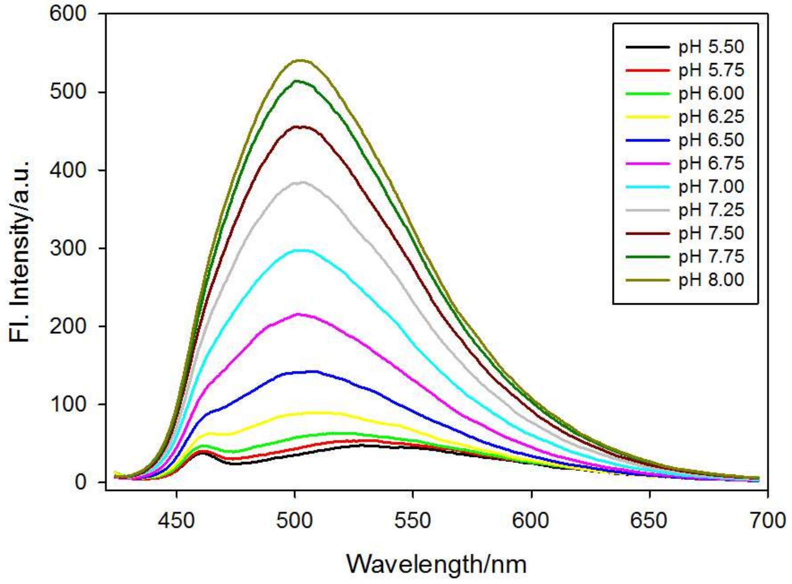

Figure S5. The emission spectra of 14tC probe at the pH range from 5.50 to 8.00.

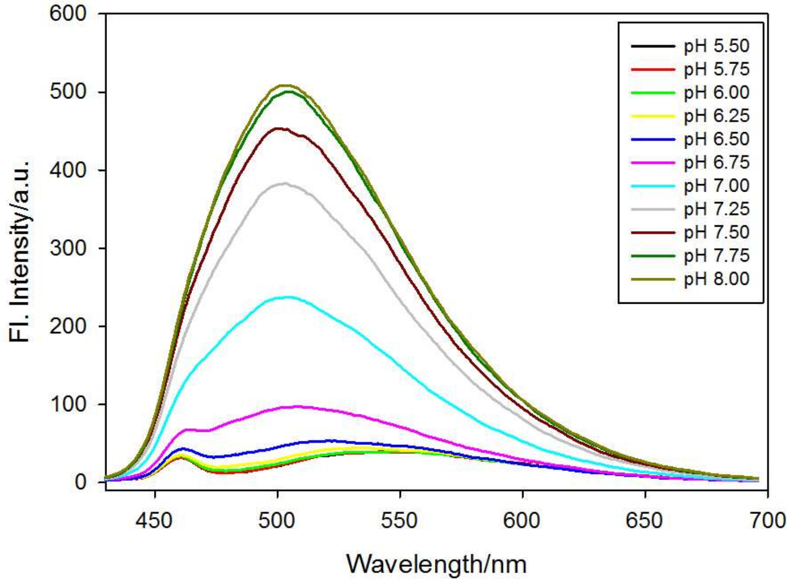

Figure S6. The emission spectra of 17tC probe at the pH range from 5.50 to 8.00.

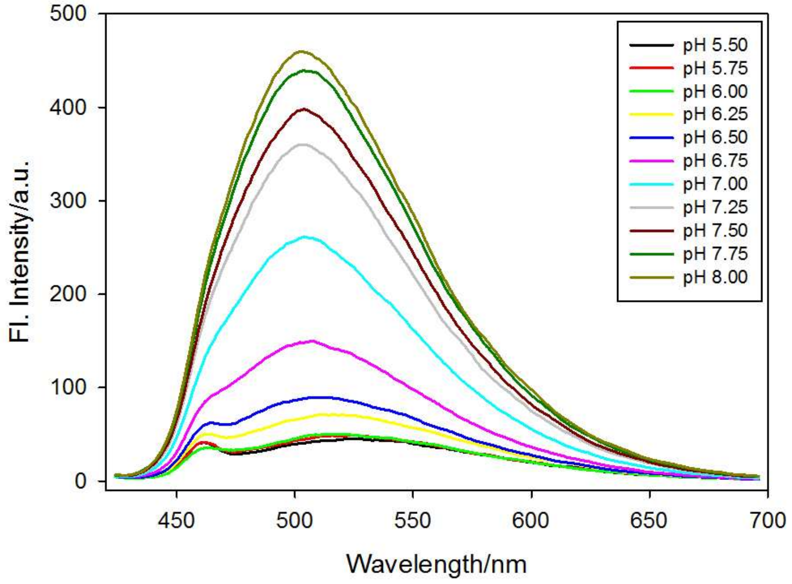

Figure S7. The emission spectra of 19tC probe at the pH range from 5.50 to 8.00.
